# Feasibility and Efficiency of Human Bone Marrow Stromal Cell Culture with Allogeneic Platelet Lysate-Supplementation for Cell Therapy against Stroke

**DOI:** 10.1155/2016/6104780

**Published:** 2016-10-20

**Authors:** Chengbo Tan, Hideo Shichinohe, Zifeng Wang, Shuji Hamauchi, Takeo Abumiya, Naoki Nakayama, Ken Kazumata, Tsuneo Ito, Kohsuke Kudo, Shigeru Takamoto, Kiyohiro Houkin

**Affiliations:** ^1^Department of Neurosurgery, Hokkaido University Graduate School of Medicine, Sapporo, Japan; ^2^Hokkaido University Hospital Clinical Research and Medical Innovation Center, Sapporo, Japan; ^3^Department of Radiology, Hokkaido University Graduate School of Medicine, Sapporo, Japan; ^4^Japanese Red Cross, Hokkaido Block Blood Center, Sapporo, Japan

## Abstract

Currently, there is increasing interest in human bone marrow stromal cells (hBMSCs) as regeneration therapy against cerebral stroke. The aim of the present study was to evaluate the feasibility and validity of hBMSC cultures with allogeneic platelet lysates (PLs). Platelet concentrates (PC) were harvested from healthy volunteers and made into single donor-derived PL (sPL). The PL mixtures (mPL) were made from three different sPL. Some growth factors and platelet cell surface antigens were detected by enzyme-linked immunosorbent assay (ELISA). The hBMSCs cultured with 10% PL were analyzed for their proliferative potential, surface markers, and karyotypes. The cells were incubated with superparamagnetic iron oxide (SPIO) agents and injected into a pig brain. MRI and histological analysis were performed. Consequently, nine lots of sPL and three mPL were prepared. ELISA analysis showed that PL contained adequate growth factors and a particle of platelet surface antigens. Cell proliferation capacity of PLs was equivalent to or higher than that of fetal calf serum (FCS). No contradiction in cell surface markers and no chromosomal aberrations were found. The MRI detected the distribution of SPIO-labeled hBMSCs in the pig brain. In summary, the hBMSCs cultured with allogeneic PL are suitable for cell therapy against stroke.

## 1. Introduction

Although studies have provided a few treatment options, ischemic stroke remains a leading cause of death and disability because of the limited regenerative capacity of the central nervous system (CNS) [1]. In recent years, the therapeutic potential of cell transplantation has been investigated in various pathological conditions of CNS [2].

Human bone marrow stromal cells (hBMSCs) are regarded as a potential cell source for ischemic stroke therapy, owing to their potential to differentiate into multiple cell lineages, their neuroprotective effects, and their ability to promote functional neural recovery of patients [3–8]. A number of experimental studies have demonstrated that transplanted BMSCs can extensively migrate towards lesions, express the phenotypes of neural cells, and improve neurological function [7, 9, 10]. Although these results are encouraging, several problems still remain unresolved, thus impeding their clinical applications. Notably, the establishment of a feasible protocol to safely expand hBMSC is a critical need. Quality, safety, and expansion are the main elements in hBMSC culture and clinical-grade expansion protocols. Particularly for clinical application, cell products must be generated in accordance with good manufacturing practice (GMP) conditions to maintain cellular quality while also minimizing the risk of adverse events.

Expansion of hBMSCs in* in vitro* culture requires the addition of supplements to the basal culture medium [11]. Fetal calf serum (FCS), an expansion supplement isolated from the clotted blood of unborn bovine fetuses, has been commonly added to cell culture mediums because of its high levels of growth stimulatory factors and low levels of growth inhibitory factors [9, 12–14]. However, there are increasing safety concerns regarding the use of FCS in clinical-scale cellular preparations because the administration of animal products to humans may theoretically cause transmittable spongiform encephalopathy (TSE) and zoonoses contamination [15–18]. Moreover, hBMSCs can internalize protein components of FCS and elicit immune reactions in the host when these foreign proteins act as antigenic substrates once transplanted [19, 20].

Human platelet lysate (PL) is a concentration of various growth factors in human platelets, obtained by lysing platelet bodies through freeze/thaw cycles or by addition of calcium chloride or thrombin activation [21]. Numerous studies have demonstrated that human PL is very effective in promoting cell expansion as well as FCS [18, 21–23]. It is known that PL includes platelet-derived growth factors (PDGFs), transforming growth factor-*β* (TGF-*β*), brain-derived neurotrophic factor (BDNF), basic fibroblast growth factor (b-FGF), vascular endothelial growth factor (VEGF), insulin-like growth factor-1 (IGF-1), and other important elements [24–26]. These factors are thought to exert important roles in promoting hBMSC expansion [21].

Now we are preparing a new clinical trial called the Research on Advanced Intervention using Novel Bone Marrow Stem Cell (RAINBOW) study, which is a phase 1 study for acute ischemic stroke patients [2]. Autologous BMSCs are cultured with allogeneic PL in the cell processing center (CPC) up to 2 cell doses: 20 million cells in the low dose group and 50 million cells in the high dose one. And BMSCs are labeled with superparamagnetic iron oxide (SPIO) for cell tracking using magnetic resonance imaging (MRI) the day before cell transplantation. They are then stereotactically transplanted around the infarct. The MRI is performed to track the donor cells until one year after the transplantation sequentially. In the present study, we aimed to evaluate the feasibility and the efficacy of hBMSC culture with allogeneic PL as GMP level and to translate the results into the RAINBOW study.

## 2. Materials and Methods

### 2.1. Isolation and Preparation of PL from Human Peripheral Blood

All experiments were performed after informed consent was obtained from healthy volunteers according to Hokkaido University's guidelines approved by the Hokkaido University Hospital's Institutional Review Board. Nine lots of platelet concentrates (PC; lots numbers 1, 2, 3, 4, 6, 7, 8, 9, and 10) were collected from nine healthy volunteers with an apheresis system for clinical use. The amount of each PC was 10 units in lots numbers 4 and 6, 20 units in numbers 8 and 10, and 15 units in others. The “unit” indicates the standard of PC preparation in Japan; that is, 10 units include 2.0–3.0 × 10^11^ cells in approximately 200 mL, 15 units include 3.0–4.0 × 10^11^ cells in approximately 250 mL, and 20 units include more than 4.0 × 10^11^ cells in approximately 250 mL. In CPC, each PC was transferred into the bags for cryopreservation (F100, Nipro, Osaka, Japan), and they were stocked in the freezer at −150°C. The PC was thawed at 37°C and transferred into the polypropylene tubes (225 mL, Ina-optika, Osaka, Japan), and then 2.5% heparin sodium (Novo-Heparin® 5000 units/5 mL, Mochida Pharmaceutical Co., Ltd., Tokyo, Japan) was added. Centrifugation with 2000 ×g for 20 min was performed twice as well incubation at 56°C for 30 min because of inactivation. Moreover, centrifugation with 500 ×g for 5 min was performed twice as well as aliquoting into polypropylene tubes (50 mL, Corning, NY) [27]. Nine lots of single donor-derived PLs (sPL; lots numbers 1, 2, 3, 4, 6, 7, 8, 9, and 10) which were derived from each PC and 3 lots of mixtures of PL (mPL) which were made from three different sPL (lots numbers 1 + 2 + 3, 4 + 6 + 7, and 8 + 9 + 10) were frozen at −80°C until being thawed just before use. We did not employ any filtration in PL preparation; filter (0.45 *μ*m, Nalgene Rapid-Flow®, Thermo Fisher Scientific, Rochester, NY) was used to reduce the impurity when 10% PL was added to the medium.

### 2.2. Measurement of Platelet Surface Antigens and Growth Factors by Enzyme-Linked Immunosorbent Assay (ELISA)

In order to clarify the residual particles of cell membranes indirectly, specific platelet cell surface antigens, CD41 (human integrin alpha-IIb ELISA kit, CUSABIO, College Park, MD) and CD61 (human integrin beta-3 ELISA kit, CUSABIO), were measured using commercially available ELISA kits in 12 PL production samples (9 sPL and 3 mPL) according to the manufacturer's instructions. Optical densities were measured by using a spectrophotometer (model 550 reader; Bio-Rad, Hercules, CA). All samples and standards were run in triplicate. The growth factor level was extrapolated from a standard curve. If any obtained data were under the mean minimum detectable dose, they were considered as nondetectable (ND) in the analysis.

Concentrations of PDGF-BB (DBB00, R&D Systems, Minneapolis, MN), TGF-*β*1 (DB100B, R&D Systems), and BDNF (DBD00, R&D Systems) in 12 PL production samples (9 sPL and 3 mPL) were also measured using commercially available ELISA kits according to the manufacturer's instructions. When the concentrations were measured, PL samples were diluted to 1 : 20 in PDGF-BB or 1 : 100 in TGF-*β*1 and BDNF.

### 2.3. Culture of hBMSCs for Cell Proliferation Assay

Two sources of hBMSCs were adopted in our present study. One was derived from a young donor by purchasing from Cell Applications Inc. (San Diego, CA). According to the manufacturer's manual, the ampoule including the frozen cells was quickly thawed in a 37°C water bath. Aseptically, the hBMSC suspension was transferred to a 15 mL tube with 10 mL of respective medium (described below) and was centrifuged at 200 ×g for 5 min. Pellets were resuspended and plated in 175 cm^2^ noncoated flasks (Easy Flask 159910; Nunc, Sigma-Aldrich, St. Louis, MO) with 25 mL of Dulbecco's modified Eagle medium (DMEM)/low glucose (D6046; Sigma-Aldrich) containing 10% preselected FCS (lot number 1355888, Gibco, Thermo Fisher Scientific, Waltham, MA) and 1% penicillin/streptomycin (P/S, Sigma-Aldrich). Cells were incubated at 5% CO_2_ at 37°C. After 2 or 3 days, nonadherent cells were washed off. The culture medium was replaced 2 or 3 times a week. After the third passage, cells were detached with a 5 min application of 0.05% Trypsin-EDTA (Gibco) at 37°C, counted, and seeded on 6-well plates (6000 cells/well) with FCS-containing DMEM (*n* = 6) or 12 lots of 10% PL-supplemented minimum essential medium alpha (*α*MEM, M0894; Sigma-Aldrich) containing gentamicin sulfate (GS, 40 *μ*g/mL; MSD, Tokyo, Japan), respectively (*n* = 3 in each PL). After 2 weeks, the cells were counted by an automated cell counter (Invitrogen, Thermo Fisher Scientific).

### 2.4. Isolation and Culture of hBMSCs in CPC

The second cell source of hBMSCs was obtained by extracting approximately 50 mL of bone marrow from a healthy volunteer. The bone marrow was brought to CPC of Hokkaido University Hospital, and the following processes were performed in the closed operation system (CPWS System Cell Processing Work Station, Panasonic Healthcare Co., Tokyo, Japan). Bone marrow mononuclear cells were isolated via density-gradient centrifugation with Ficoll-Hypaque (Pharmacia, Uppsala, Sweden), and 1.1 × 10^7^ cells were plated in a 75 cm^2^ noncoated flasks (Easy Flask 156499; Nunc) with 15 mL of *α*MEM with 10% mPL and 40 *μ*g/mL GS. After 48 h, nonadherent cells were removed by changing the medium. The culture medium was replaced 2 or 3 times a week. The hBMSCs were passed three times for the subsequent transplantation. In order to label the cells for MRI tracking, 1 *μ*L/mL Ferucarbotran (27.9 *μ*g Fe/mL, Resovist®, Fujifilm RI Pharma Co., Ltd., Tokyo, Japan), a SPIO agent, was added into the culture medium to be incubated with hBMSCs 24 h before cell injection. SPIO-labeled hBMSCs in flasks were lifted using 4 mL TrypLe Select® (a recombinant trypsin substitute, Gibco) and incubated for 5 min. After fully agitating, cell suspensions were transferred into test tubes and centrifuged at 800 ×g, 5 min at 15°C. The supernatant was decanted and the cells were gently resuspended by Artcereb® (the irrigation and perfusion solution for cerebrospinal surgery; Otsuka Pharmaceutical Factory, Inc., Naruto, Japan) to 5 × 10^7^ cells/mL. In order to analyze SPIO-positive hBMSCs, 600 *μ*L/well cell suspensions were seeded on a fibronectin-coated four-well (1.7 cm^2^ per well) chamber slide. After 24 h, the medium was discarded and the cells on the culture slide were rinsed twice with phosphate buffered saline (PBS). The cells were fixed with 4% acetone for 3 min and then immersed in PBS for 10 min. Subsequently, the slide was stained by Turnbull's Blue method and counted to analyze the concentration of SPIO-labeled hBMSCs.

### 2.5. Flow Cytometric Analysis

Flow cytometric analysis was performed to evaluate the surface markers of hBMSCs. The hBMSCs cultured with PL in CPC were suspended with PBS containing 3% FCS. They were incubated with either a mouse monoclonal antibody against human CD19 (R&D Systems; dilution, 1 : 100), CD44 (R&D Systems; 1 : 100), CD45 (R&D Systems; 1 : 100), CD90 (R&D Systems; 1 : 100), CD105 (R&D Systems; 1 : 100), CD106 (R&D Systems; 1 : 100), CD146 (R&D Systems; 1 : 100), CD166 (R&D Systems; 1 : 100), or each mouse isotypic control for 30 min on ice. Cell suspensions were then incubated with Alexa Fluor 488-conjugated secondary antibodies (Molecular Probes, Thermo Fisher Scientific; 1 : 200) for 30 min on ice. Flow cytometric analysis was performed after two washes using a cytometer (Attune® Acoustic Focusing Cytometer, Applied Biosystems, Thermo Fisher Scientific). Live events (10,000) were acquired for analysis.

### 2.6. Karyotype Analysis

Karyotype analysis was performed by Q-banding using conventional methods (Nihon Gene Research Laboratories Inc., Sendai, Japan). The hBMSCs obtained by purchasing from Cell Applications Inc. (San Diego, CA) were prepared as mentioned above. The cells were cultured with autologous PL supplementation and passed twice before the analysis. The cells were divided into two groups about pretreatment; thus, one was treated with the synchronous culture using thymidine, while the other was not treated with it. The cells in both groups were incubated in quinacrine mustard dihydrochloride for 10 min and then immersed in PBS. They were stained with Hoechst 33258 for 10 min and immersed in distilled water. They were mounted on the slides with an antifade reagent.

### 2.7. Cell Injection into Decapitated Pig Brain Parenchyma and MR Imaging

The SPIO-labeled hBMSCs were injected into the striatum of a decapitated pig brain. A burr hole was made 3 cm left of the bregma using a small dental drill. A cell injection needle (Mizuho Co., Tokyo, Japan) attached to a syringe was inserted 4 cm into the brain parenchyma. Then, 300 *μ*L of the cell suspension (5 × 10^4^ cells/*μ*L) was injected over 5 min. After injection, the needle was left* in situ* for 5 min to avoid leakage of the injected fluid through the needle tract [28].

All MRI data were acquired using a clinical MR scanner (TRILLIUM OVAL, Hitachi, Tokyo, Japan). Quantitative susceptibility mapping (QSM) images were acquired by the use of an RSSG EPI sequence. The sequence parameters were repetition time (TR) = 30 msec, echo time (TE) = 15 msec, flip angle = 15°, number of acquisition (AC) = 0, matrix = 512 × 512, and slice thickness = 1.2 mm.

### 2.8. Histological Analysis

The decapitated pig brain in which SPIO-hBMSCs were injected for MR imaging was used for histological analysis. The day after cell injection, the brain was removed from the skull and stored in 4% paraformaldehyde for one week. It was then sliced and embedded in Tissue Freezing Medium OCT Compound (Sakura Finetek Japan Co., Ltd., Tokyo, Japan) and quickly frozen in liquid nitrogen. Frozen sections of 12 *μ*m thickness were mounted on silane-coated glass slides. During immunohistochemical analysis, sections were treated with peroxidase blocking reagent (Dako Japan, Tokyo, Japan) and then incubated with 1% block ace solution (DS Pharma Biomedical, Osaka, Japan) for 30 min. Sections were treated with a primary mouse anti-human nuclei monoclonal antibody (MAB1281; 1 : 100, Chemicon, Temecula, California, USA) at 4°C overnight. Dako EnVision+ Kit (Dako Japan) was then applied for 1 h. The DAB Substitute Kit (Dako Japan) was also employed according to the manufacturer's instructions. After staining, sections were dipped into 10% ammonium sulfide solution (Sigma-Aldrich) overnight, followed by incubation with compound solution mixed in 20% potassium ferricyanide (Wako, Osaka, Japan) and 1% HCL solution for 20 min. Nuclear fast red solution was used for counterstaining. Another section was treated with MAB1281 (1 : 100) alone and then colored with Dako EnVision+ kit and DAB Substitute Kit. Hematoxylin was applied for counterstaining.

For fluorescent immunohistochemistry, after blocking nonspecific reactions, sections were treated with MAB1281 (1 : 100 dilution) as the primary antibody at 4°C overnight followed by incubation with Alexa Fluor 594 Goat Anti-mouse IgG (H + L) Antibody (1 : 200, Life Technologies, Thermo Fisher Scientific) as the secondary antibody at room temperature for 1 h. The fluorescence emitted was observed through each appropriate filter on a fluorescence microscope (BX51, Olympus, Tokyo, Japan) and was digitally photographed using a cooled CCD camera (model VB-6000/6010, Keyence, Osaka, Japan).

## 3. Results

### 3.1. Residues of Cell Membrane in PL

**Figure 1:**
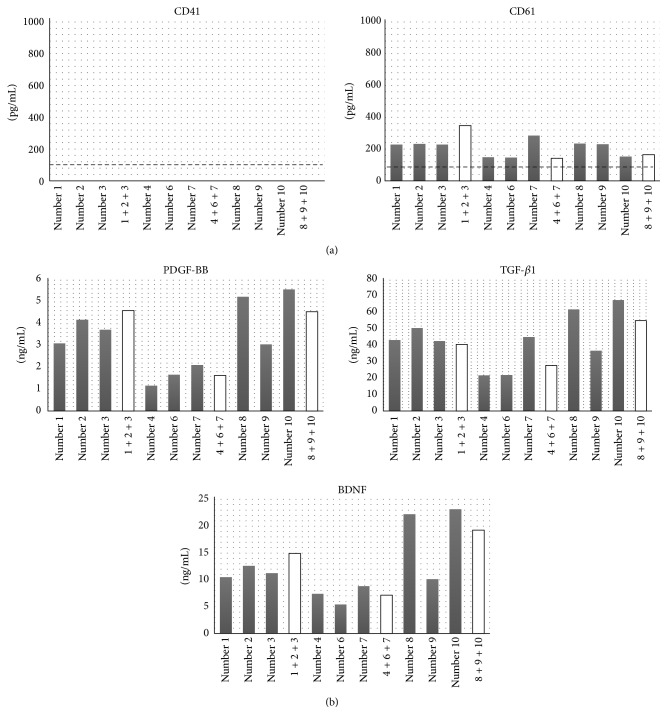
(a) shows ELISA analysis for cell surface antigens of platelets (left: CD41, right: CD61). The broken lines indicate each mean minimum detectable dose. (b) shows the measurement of growth factors in PL samples. Each graph indicates the concentration of human PDGF-BB, TGF-*β*1, and BDNF in the 12 lots of fresh PL samples, respectively. Gray bars: single donor-derived PL (sPL); white bars: mixtures type of PL (mPL).

ELISA analysis demonstrated that the platelet cell surface antigen CD41 of all PL samples was not detected (the lower limitation of detect range: 156 pg/mL). CD61 was detected in the all samples; however, the mean amount was extremely small (210 ± 63 pg/mL; mean ± SD, Figure 1(a)).

### 3.2. Growth Factors Contained in PL

The mean concentrations of human PDGF-BB, TGF-*β*1, and BDNF were 3.36 ± 2.20 ng/mL, 44.9 ± 23.0 ng/mL, and 14.3 ± 8.9 ng/mL in all PL samples, respectively (Figure 1(b)). The concentration in each PL seemed to be correlated among the growth factors and there was a big difference between each sample, especially sPL, for every growth factor. When the data were analyzed for the platelet units in original PC, we found that the amount of these growth factors correlated with platelet units in original PC (Figure 3(a)). Among mPL, there was a relatively small difference in concentrations because they were balanced by mixtures of 3 different sPL (Figure 1(b)).

### 3.3. Cell Proliferative Potential of PL

**Figure 2:**
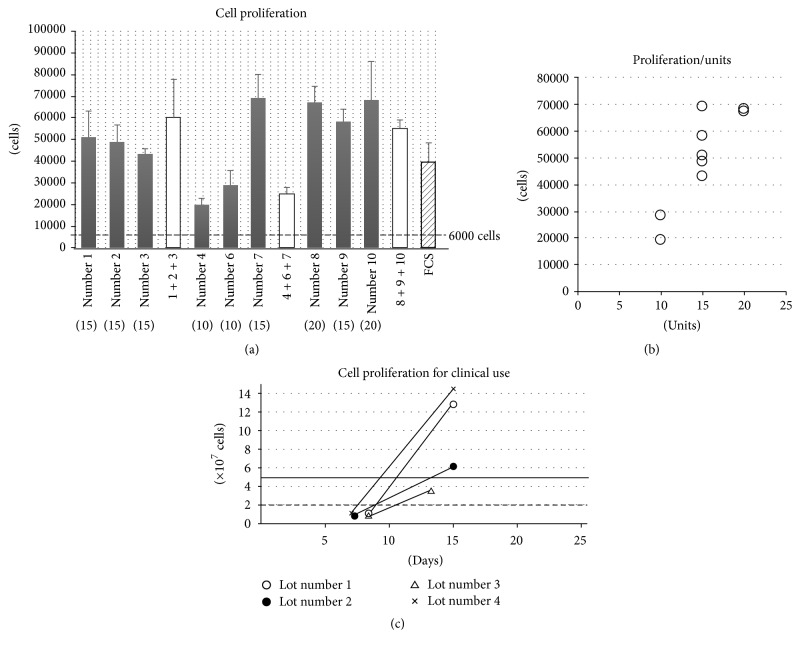
Cell proliferative potential for PL or FCS medium. (a) shows the quantity of cultured cells in PL- or FCS-supplemented culture medium. Gray bars: cells cultured with sPL; white bars: cells cultured with mPL; striped bar: the cells cultured with FCS. Error bars: SD. Broken line: the quantity of seeded cells on a well (6000 cells). (b) shows the quantity of cultured cells in PL-supplemented culture medium. *x*-axis: numbers of the platelet units in original PC. (c) showed the cell proliferation when cultured in CPC as a simulation of the clinical trials, the RAINBOW study. Broken line: 20 million cells as the target in the low dose group. Solid line: 50 million cells as the target in the high dose group.

**Figure 3:**
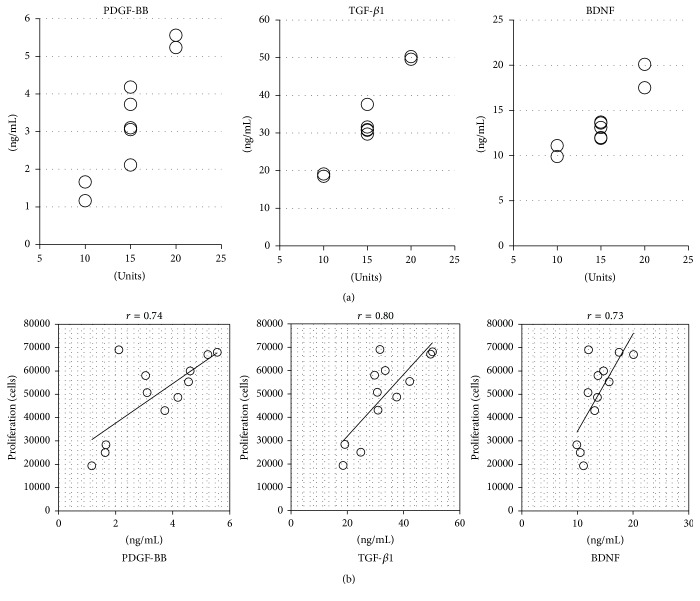
Cell proliferative potential and growth factors. (a) shows the concentration of each growth factor (left: PDGF-BB; center: TGF-*β*1; right: BDNF). *x*-axis: numbers of the platelet units in original PC. (b) shows the correlation between cell proliferative potential and the concentration of each growth factor (left: PDGF-BB; center: TGF-*β*1; right: BDNF).

Cell proliferation assays demonstrated the expansive capacity of hBMSCs with 12 lots of PL-supplemented *α*MEM (9 sPL and 3 mPL) or FCS-supplemented DMEM. Two weeks after cell seeding, a distinct difference existed between each quantity of cell proliferation (Figure 2(a)). Compared with the cell proliferation in FCS medium, most of the PL medium had the equivalent or much higher expansive ability. However, the sPL derived from 10 unit-PC, which includes 2.0–3.0 × 10^11^ cells in approximately 200 mL, were lower than FCS (number 4 and number 6). Moreover, one of the mPL, number 4 + 6 + 7, also had a lower proliferative potential because it was made of number 4 and number 6 in sPL (Figure 2(a)). Thus, when the data were analyzed regarding the platelet units in original PC, we found a correlation between the unit number and proliferative potential (Figure 2(b)). Moreover, the proliferative potential was positively correlated with the concentrations of PDGF-BB (*r* = 0.74), TGF-*β*1 (*r* = 0.80), and BDNF (*r* = 0.73, Figure 3(b)).

The hBMSCs derived from 4 healthy volunteers (lots numbers 1, 2, 3, and 4) were cultured in CPC as a simulation of the RAINBOW study (Figure 2(c)). The cells in each lot were passed first on day 7 or 8 after the culture. The cells in 3 lots were passed second on day 15, and the cell numbers in each lot reached over 5 × 10^7^ cells which is the target in the high dose group. On the other hand, the cells in lot number 3 were passed second on day 13 and the cell numbers reached 3.5 × 10^7^ cells. The cell numbers were over 20 million which is the target in the low dose group (Figure 2(c)). Thus, the cell numbers could almost reach the target in our clinical trials (low dose: 4/4, high dose: 3/4) with 2 passages for 2 weeks.

### 3.4. Surface Marker of hBMSC

**Figure 4:**
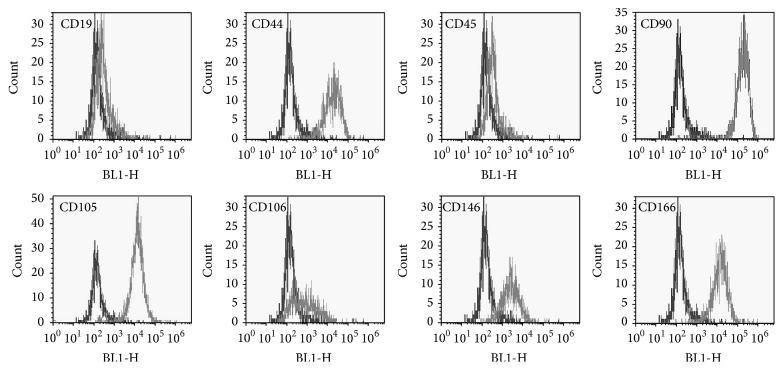
Flow cytometric analysis of the surface markers. Gray lines: each specific antibody (CD44, CD90, CD105, CD106, CD146, CD166, CD19, and CD45); black lines: each isotopic antibody.

Flow cytometric analysis was performed to assay the surface markers of hBMSCs cultured with PL-supplemented *α*MEM. These cells expressed CD44 (96.5 ± 2.5%), CD90 (97.3 ± 3.5%), CD105 (98.5 ± 0.6%), CD106 (51.5 ± 14.8%), CD146 (36.8 ± 18.1%), and CD166 (97.8 ± 1.5%), while there was absence of CD19 (2.8 ± 1.7%) and CD45 (1.8 ± 0.5%) (*n* = 4 in each, Figure 4).

### 3.5. Karyotype of hBMSC

**Figure 5:**
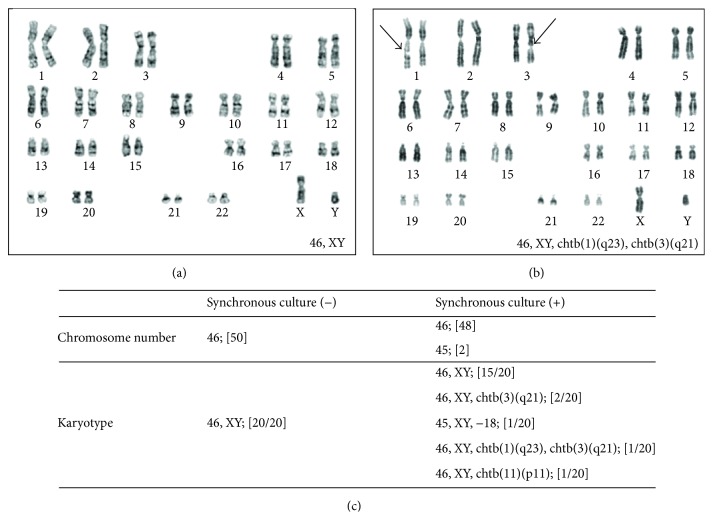
Karyotype analysis. (a) and (b) show the representative photomicrographs of karyotype ((a) no pretreatment and (b) the pretreatment with the synchronous culture). Arrows on (b): the location of the chromatid breaks (chtb). Table (c) shows the summary of karyotype analysis.

The hBMSCs were cultured with 10% PL-supplemented *α*MEM, and the karyotype was analyzed after second passage. Karyotype analysis showed that hBMSCs had normal chromosome number and karyotype when they did not have synchronous culture as pretreatment (Figure 5(a)). But the pretreatment with the synchronous culture using thymidine influenced the analysis to produce artifact (Figure 5(b)). Thus the synchronous culture caused abnormal chromosome number (45; [2/50]) and karyotype (46, XY, chtb(3)(q21); [2/20], 45, XY, −18; [1/20], 46, XY, chtb(1)(q23), chtb(3)(q21); [1/20], 46, XY, chtb (11)(p11); [1/20]) as artifact (Figure 5(c)).

Moreover, these cells were checked for soft agar colony forming test and* in vivo* tumorigenicity test with nude rats as preclinical test, and no tumorigenicity was shown (data was not shown).

### 3.6. SPIO-Labeled hBMSC and QSM MRI

**Figure 6:**
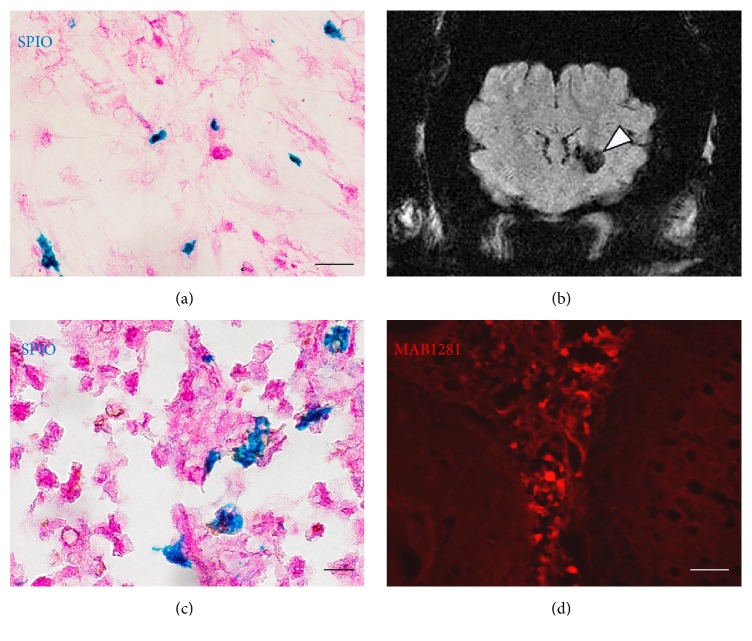
MRI and histological analysis of pig's brain with SPIO-labeled hBMSC injection. (a) shows the photomicrographs of the cultured SPIO-hBMSCs after Turnbull's Blue staining (blue: SPIO). Scale bars: 50 *μ*m. (b) represents QSM MR images. Arrowheads indicate the signal extinctions caused by the bolus of SPIO-labeled hBMSCs in the left striatum. (c) and (d) display representative photomicrographs of the region around the SPIO-hBMSC injection after Turnbull's Blue staining (blue: SPIO, (c)) and fluorescence immunostaining (red: MAB1281, (d)). Scale bars: 20 *μ*m (c) and 50 *μ*m (d).

Turnbull's Blue staining demonstrated that approximately 34% of the PL-cultured hBMSCs were labeled with SPIO 24 h after incubation with SPIO nanoparticles (Figure 6(a)). The MRI for clinical use could visualize the bolus of SPIO-hBMSCs engrafted in the decapitated pig brain. The cell bolus showed a strong signal loss when imaged with QSM methods (Figure 6(b)). Histological analysis clearly revealed that some SPIO-positive cells (Figure 6(c)) or human cells (Figure 6(d)) were found around the injection region. These findings were consistent with the results of MRI.

## 4. Discussion

In the present study, we harvested 12 lots of human PLs as growth supplements instead of FCS. ELISA analysis showed that PL contained sufficient growth factors to nourish hBMSCs and very small amount of platelet surface antigens. Although the PL had equivalent or higher cell proliferation capacity compared with FCS, there was no contradiction to BMSC for cell surface markers and no abnormal karyotype in hBMSC-PL. About 2 weeks, the cell numbers could reach up to 2 × 10^7^ cells which is the target in our clinical trials in every lot. When SPIO-labeled hBMSCs were injected into the pig brain, MRI could detect their distribution the same as histological analysis.

When PL products are made from human PC in accordance with GMP, we noticed the existence of fragments of platelet membranes as residue materials. Instead of fragments of platelet membranes, we analyzed the platelet surface antigens in PL products because it is difficult to detect the residual fragments themselves. In the present study, we detected a very small amount of CD61, but not CD41. The findings suggested that the amount of CD61 in PL was useful for quality control of residue materials when produced on GMP level. In fact, the mean content of CD61 in PL was 210 ± 63 pg/mL (the lower limitation of detect range: 125 pg/mL) in our present data., so the presence of CD61 is unable to be detected in culture medium because only 10% PL was added to the medium and filtering was employed to reduce the impurity. This finding demonstrated that our PL products held adequate safety and quality for clinical application.

Various studies have indicated that growth factors such as PDGF-BB, TGF-*β*1, and BDNF play a prominent role in BMSC culture. PDGF-BB can elicit a mitogenic response from BMSCs and stimulates these cells to proliferate [29–31]. It has also been demonstrated that TGF-*β*1 could stimulate the proliferation of undifferentiated MSC [31, 32]. In contrast, our previous report showed that the concentration of human BDNF, which was derived from PL, markedly decreased in the medium after hBMSC culture. These results strongly suggested that the cultured hBMSCs may consume human BDNF for their survival and proliferation [17]. In the present study, we found that the content of these growth factors in each PL was relevant to the amount of platelets in original PC. Furthermore, the ability of cell expansion correlated with the contents of the growth factors in each PL. When the contents of platelets in original PC reached more than 15 units, the cell proliferating potential of PL was equivalent or much higher compared with the FCS. This indicated that the PL supplement contains adequate essential growth factors and nutrition as well as FCS for the expansion of hBMSC. When made in accordance with GMP, however, the findings suggested that we could check the contents of a growth factor, PDGF-BB, TGF-*β*1, or BDNF as a quality control of PL products instead of the potential for cell expansion. Moreover, because there was a smaller difference among mPL compared with sPL regarding the contents of growth factors, pooled PL should be useful in mass production.

It is well known that hBMSCs express CD44, CD90, CD105, CD106, CD146, and CD166 but not CD14, CD19, CD34, and CD45 [33–35]. In our previous study, no significant differences were observed between hBMSCs cultured using 5% PL and using 10% FCS [17]. In the present study, although the concentration of PL was changed to 10%, we confirmed that the hBMSCs were identical for surface markers. Thus, our findings demonstrate that 10% PL-cultured hBMSCs are reliable for clinical application as well as 5% PL-cultured cells. In the productions of hBMSC products, moreover, the present results suggested that CD44, CD90, CD105, and CD166, but not CD106 or CD146, should be suitable positive makers for the specific test, because of their high percentage and low SD.

In our present study, karyotype analysis showed that 10% PL-cultured hBMSCs had normal chromosome number and karyotype after 2 passages. Because the commercial cells had 3 passages before usage, in fact the karyotype analysis was done after 5 passages. We have to urge caution about the artifact as abnormal karyotype due to the synchronous culture using thymidine. Although the mechanism is unclear, it suggested that the pretreatment might be unsuitable for karyotype analysis with hBMSC.

In our new clinical trials, RAINBOW study, the subjects are acute ischemic stroke patients. Autologous bone marrow is obtained 2 weeks after the stroke onset. And then BMSCs are cultured with allogeneic PL in CPC up to 2 cell doses: 20 million cells in the low dose group and 50 million cells in the high dose one. In the present study, the cell numbers could reach up to 5 × 10^7^ cells in only 3 lots, though the numbers could increase over 2 × 10^7^ cells in every 4 lots 2 weeks after the start of culture. So we decided to translate the results to the protocol of RAINBOW study. Thus, in the patient allocated to the high dose group, the patient would be shifted to the low dose group if the cell numbers could not reach up to 5 × 10^7^ cells in a period.

In addition, hBMSCs were visualized in a decapitated pig brain. Cell labeling with SPIO was employed to track donor cells in the host brain by means of MRI. Cultured hBMSCs can uptake SPIO nanoparticles into their cytoplasm when the particles are added to the culture medium [7]. In the present study, Turnbull's Blue staining analysis showed that 34% hBMSCs in a chamber slide contained SPIO nanoparticles. Because SPIO nanoparticles have clearly detectable signal extinctions, SPIO-labeled cells were easily tracked anatomically with QSM MR images [7]. Histological analysis gained the same hBMSC distribution compared with MRI. Our previous study identified the long term safety of SPIO-labeled BMSCs [7]. Thus, we believe that MRI cell tracking with SPIO based labeling agents is a good resource to monitor cell distribution after hBMSC transplantation. We hope this technology can be used for cell therapy in clinical applications.

In conclusion, our present findings demonstrate that hBMSCs cultured with allogeneic PL may be valuable, feasible, and safe for cell therapy against ischemic stroke.
